# Supplementary material for: Knowledge and attitudes of university staff toward organ donation: a cross-sectional study in Oman
Source: PeerJ. 2025 Oct 6;13:e20133. doi: 10.7717/peerj.20133 (PMC12510254; doi:10.7717/peerj.20133)
Supplement: Supplemental Information 1 [file peerj-13-20133-s001.docx]

| **Sociodemographic characteristics** | | **Knowledge about organ donation** | | | | **p-value** | **X^2^ (df)** |
| --- | --- | --- | --- | --- | --- | --- | --- |
|  |  | **Poor** | | **Good** | |  |  |
|  |  | Number | Percent | Number | Percent |  |  |
| **Sex** | Male | 63 | 46.0 | 74 | 54.0 | 0.001 | 16.780 (1) |
|  | Female | 62 | 25.0 | 186 | 75.0 |  |  |
| **Age groups** | 18-29 | 17 | 31.5 | 37 | 68.5 | 0.576 | 1.985 (3) |
|  | 30-41 | 62 | 30.8 | 139 | 69.2 |  |  |
|  | 42-53 | 38 | 33.6 | 75 | 66.4 |  |  |
|  | 54-65 | 8 | 47.1 | 9 | 52.9 |  |  |
| **Marital status** | Single | 22 | 29.3 | 53 | 70.7 | 0.669 | 0.803 (2) |
|  | Married | 100 | 33.6 | 198 | 66.4 |  |  |
|  | Divorced | 3 | 25.0 | 9 | 75.0 |  |  |
| **Academic degree** | Undergraduate or less | 82 | 33.6 | 162 | 66.4 | 0.607 | 0.265 (1) |
|  | Postgraduate | 43 | 30.5 | 98 | 69.5 |  |  |
| **Job title** | Administrative staff | 73 | 38.4 | 117 | 61.6 | 0.001 | 17.503 (3) |
|  | Medical staff | 2 | 5.3 | 36 | 94.7 |  |  |
|  | Technical staff | 36 | 35.3 | 66 | 64.7 |  |  |
|  | Academic staff | 14 | 25.5 | 41 | 74.5 |  |  |
| **Number of working years** | 1-11 | 57 | 29.5 | 136 | 70.5 | 0.002 | 11.644 (2) |
|  | 12-23 | 40 | 28.8 | 99 | 71.2 |  |  |
|  | 24-35 | 28 | 52.8 | 25 | 47.2 |  |  |
