# Supplementary material for: Knowledge and attitudes of university staff toward organ donation: a cross-sectional study in Oman
Source: PeerJ. 2025 Oct 6;13:e20133. doi: 10.7717/peerj.20133 (PMC12510254; doi:10.7717/peerj.20133)
Supplement: Supplemental Information 2 [file peerj-13-20133-s002.docx]

| **Sociodemographic characteristics** | | **Attitude about organ donation** | | | | **p-value** | **X^2^ (df)** |
| --- | --- | --- | --- | --- | --- | --- | --- |
|  |  | **Negative** | | **Positive** | |  |  |
|  |  | **Number** | **Percent** | **Number** | **Percent** |  |  |
| Sex | Male | 92 | 67.7 | 45 | 32.8 | 0.147 | 2.101 (1) |
|  | Female | 148 | 59.7 | 100 | 40.3 |  |  |
| Age groups | 18-29 | 25 | 46.3 | 29 | 53.7 | 0.065 | 7.228 (3) |
|  | 30-41 | 133 | 66.2 | 68 | 33.8 |  |  |
|  | 42-53 | 71 | 62.8 | 42 | 37.2 |  |  |
|  | 54-65 | 11 | 64.7 | 6 | 35.3 |  |  |
| Marital status | Single | 39 | 52.0 | 36 | 48.0 | 0.029 | 7.095 (2) |
|  | Married | 196 | 65.8 | 102 | 34.2 |  |  |
|  | Divorced | 5 | 41.7 | 7 | 58.3 |  |  |
| Academic degree | Undergraduate or less | 158 | 64.8 | 86 | 35.2 | 0.198 | 1.657 (1) |
|  | Postgraduate | 82 | 58.2 | 59 | 41.8 |  |  |
| Job title | Administrative staff | 132 | 69.5 | 58 | 30.5 | 0.001 | 18.386 (3) |
|  | Medical staff | 15 | 39.5 | 23 | 60.5 |  |  |
|  | Technical staff | 67 | 65.7 | 35 | 34.3 |  |  |
|  | Academic staff | 26 | 47.3 | 29 | 52.7 |  |  |
| Number of working years | 1-11 | 120 | 50.0 | 73 | 50.3 | 0.147 | 3.836 (2) |
|  | 12-23 | 81 | 33.7 | 58 | 40.0 |  |  |
|  | 24-35 | 39 | 16.3 | 14 | 9.7 |  |  |
