# Supplementary material for: Knowledge and attitudes of university staff toward organ donation: a cross-sectional study in Oman
Source: PeerJ. 2025 Oct 6;13:e20133. doi: 10.7717/peerj.20133 (PMC12510254; doi:10.7717/peerj.20133)
Supplement: Supplemental Information 3 [file peerj-13-20133-s003.docx]

| **What supports you to donate your organs?** | **Number** | **Percent** |
| --- | --- | --- |
| By donating an organ, you are saving a life. | 259 | 67.30 |
| Islamic religion allows me to do so. | 180 | 46.80 |
| I would only become a donor for someone dear to me. | 91 | 23.60 |
| I really want to help a fellow person. | 73 | 19.00 |
| The mass media has had a positive effect on me about becoming a donor. | 44 | 11.40 |
| I have been affected by a family member or a friend that is a donor. | 34 | 8.80 |
| I'm against organ donation. | 22 | 5.70 |
| I have been sensitized by a family member or friend that needed a transplant. | 15 | 3.90 |
| Others. | 11 | 2.90 |
| I need the money. | 8 | 2.10 |
