# Supplementary material for: Knowledge and attitudes of university staff toward organ donation: a cross-sectional study in Oman
Source: PeerJ. 2025 Oct 6;13:e20133. doi: 10.7717/peerj.20133 (PMC12510254; doi:10.7717/peerj.20133)
Supplement: Supplemental Information 4 [file peerj-13-20133-s004.docx]

|  | | **Saving a Life** | | | **Islamic allows** | | |
| --- | --- | --- | --- | --- | --- | --- | --- |
| **Sociodemographic characteristics** | | **N (%)** | **p-value** | **X^2^ (df)** | **N (%)** | **p-value** | **X^2^ (df)** |
| Sex | Male | 87 (33.6%) | 0.257 | 1.286 (1) | 60(33.3) | 0.387 | 0.747(1) |
|  | Female | 172 (66.4%) |  |  | 120(66.7) |  |  |
| Age groups | 18-29 | 42 (16.2%) | 0.224 | 4.378 (3) | 18(10) | 0.044 | 8.111(3) |
|  | 30-41 | 134 (51.7%) |  |  | 91(50.6) |  |  |
|  | 42-53 | 74 (28.6%) |  |  | 60(33.3) |  |  |
|  | 54-65 | 9 (3.5%) |  |  | 11(6.1) |  |  |
| Marital status | Single | 57 (22%) | 0.224 | 2.995 (2) | 31(17.2) | 0.335 | 2.187(2) |
|  | Married | 194 (74.9%) |  |  | 145(80.6) |  |  |
|  | Divorced | 8 (3.1%) |  |  | 4(2.2) |  |  |
| Academic degree | Undergraduate or less | 161 (62.2%) | 0.451 | 0.569 (1) | 106(58.9) | 0.087 | 2.933(1) |
|  | Postgraduate | 98 (37.8%) |  |  | 74(41.1) |  |  |
| Job title | Administrative staff | 116 (44.8%) | 0.046 | 7.997 (3) | 88(48.9) | 0.645 | 1.662(3) |
|  | Medical staff | 31 (12%) |  |  | 21(11.7) |  |  |
|  | Technical staff | 73 (28.2%) |  |  | 48(26.7) |  |  |
|  | Academic staff | 39 (15.1%) |  |  | 23(12.8) |  |  |
| Number of working years | 1-11 | 137 (52.9%) | 0.158 | 3.694 (2) | 80(44.4) | 0.102 | 4.575(2) |
|  | 12-23 | 92 (35.5%) |  |  | 71(39.4) |  |  |
|  | 24-35 | 30 (11.6%) |  |  | 29(16.1) |  |  |
