# Supplementary material for: Knowledge and attitudes of university staff toward organ donation: a cross-sectional study in Oman
Source: PeerJ. 2025 Oct 6;13:e20133. doi: 10.7717/peerj.20133 (PMC12510254; doi:10.7717/peerj.20133)
Supplement: Supplemental Information 5 [file peerj-13-20133-s005.docx]

| **What are the reasons that make you refuse organ donation?** | **Number** | **Percent** |
| --- | --- | --- |
| I’m still not aware or decided regarding organ donation. | 176 | 45.70 |
| I have no objection for organ donation. | 157 | 40.80 |
| I’m afraid. | 101 | 26.20 |
| The health system in Oman is not yet equipped for organ donation services. | 73 | 19.00 |
| I do not believe that organ donation would be used correctly. | 40 | 10.40 |
| Against Islamic religion. | 19 | 4.90 |
| Others. | 9 | 2.30 |
| I find it irrelevant and am not really concerned about the matter. | 8 | 2.10 |
