# Supplementary material for: Knowledge and attitudes of university staff toward organ donation: a cross-sectional study in Oman
Source: PeerJ. 2025 Oct 6;13:e20133. doi: 10.7717/peerj.20133 (PMC12510254; doi:10.7717/peerj.20133)
Supplement: Supplemental Information 6 [file peerj-13-20133-s006.docx]

|  | | **I'm Afraid** | | | **Against Islamic Religion** | | |
| --- | --- | --- | --- | --- | --- | --- | --- |
| **Sociodemographic characteristics** | | **N (%)** | **p-value** | **X^2^ (df)** | **N (%)** | **p-value** | **X^2^ (df)** |
| Sex | Male | 33 (32.4%) | 0.427 | 0.632 (1) | 6 (31.6%) | 0.708 | 0.140 (1) |
|  | Female | 69 (67.6%) |  |  | 13 (68.4%) |  |  |
| Age groups | 18-29 | 23 (22.5%) | 0.005 | 12.903 (3) | 1 (5.3%) | 0.292 | 3.728 (3) |
|  | 30-41 | 56 (54.9%) |  |  | 8 (42.1%) |  |  |
|  | 42-53 | 21 (20.6%) |  |  | 9 (47.4%) |  |  |
|  | 54-65 | 2 (2%) |  |  | 1 (5.3%) |  |  |
| Marital status | Single | 24 (23.5%) | 0.384 | 1.913 (2) | 5 (26.3%) | 0.104 | 4.524 (2) |
|  | Married | 76 (74.5%) |  |  | 12 (63.2%) |  |  |
|  | Divorced | 2 (2%) |  |  | 2 (10.5%) |  |  |
| Academic degree | Undergraduate or less | 76 (74.5%) | 0.006 | 7.410 (1) | 13 (68.4%) | 0.64 | 0.219 (1) |
|  | Postgraduate | 26 (25.5%) |  |  | 6 (31.6%) |  |  |
| Job title | Administrative staff | 60 (58.8%) | 0.008 | 11.851 (3) | 9 (47.4%) | 0.331 | 3.423 (3) |
|  | Medical staff | 8 (7.8%) |  |  | 1 (5.3%) |  |  |
|  | Technical staff | 29 (28.4%) |  |  | 8 (42.1%) |  |  |
|  | Academic staff | 5 (4.9%) |  |  | 1 (5.3%) |  |  |
| Number of working years | 1-11 | 62 (60.8%) | 0.023 | 7.539 (2) | 6 (31.6%) | 0.23 | 2.944 (2) |
|  | 12-23 | 32 (31.4%) |  |  | 10 (52.6%) |  |  |
|  | 24-35 | 8 (7.8%) |  |  | 3 (15.8%) |  |  |
