# Supplementary material for: Knowledge and attitudes of university staff toward organ donation: a cross-sectional study in Oman
Source: PeerJ. 2025 Oct 6;13:e20133. doi: 10.7717/peerj.20133 (PMC12510254; doi:10.7717/peerj.20133)
Supplement: Supplemental Information 7 [file peerj-13-20133-s007.docx]

| **Information sources** | **Knowledge** | | **p-value** | **X^2^ (df)** | **Attitude** | | **p-value** | **X^2^ (df)** |
| --- | --- | --- | --- | --- | --- | --- | --- | --- |
|  | **Good**  **N (%)** | **Poor**  **N (%)** |  |  | **Positive**  **N (%)** | **Negative**  **N (%)** |  |  |
| University | 56 (66.7%) | 28 (33.3%) | 0.952 | 0.004 (1) | 34 (40.5%) | 50 (59.5%) | 0.635 | 0.225(1) |
| Health care facilities. | 87 (70.2%) | 37 (29.8%) | 0.520 | 0.413 (1) | 47 (37.9%) | 77 (62.1%) | 1.00 | 0.000(1) |
| Internet/online source/ social network. | 208 (72.7%) | 78 (27.3%) | 0.001 | 12.783 (1) | 114 (39.9%) | 172 (60.1%) | 0.164 | 1.939(1) |
| Newspaper. | 38 (73.1%) | 14 (26.9%) | 0.448 | 0.576(1) | 18 (34.6%) | 34 (65.4%) | 0.739 | 0.111(1) |
| Posters. | 47 (75.8%) | 15 (24.2%) | 0.170 | 1.879(1) | 33 (53.2%) | 29 (46.8%) | 0.009 | 6.855(1) |
| Organ donation promotion campaigns. | 77 (72.6%) | 29 (27.4%) | 0.231 | 1.435(1) | 51 (48.1%) | 55 (51.9%) | 0.013 | 6.204(1) |
| Family, Friends and, Colleagues. | 84 (74.3%) | 29 (25.7%) | 0.086 | 2.952(1) | 48 (42.5%) | 65 (57.5%) | 0.254 | 1.303(1) |
| Radio/ Television. | 100 (69.4%) | 44 (30.6%) | 0.612 | 0.257(1) | 56 (38.9%) | 88 (61.1%) | 0.783 | 0.076(1) |
| Others | 3 (50%) | 3 (50%) | 0.628 | 0.235(1) | 3 (50%) | 3 (50%) | 0.838 | 0.042(1) |
